# Effects of Neonatal Fluvoxamine Administration on the Physical Development and Activity of the Serotoninergic System in White Rats

**Published:** 2014

**Authors:** N. Yu. Glazova, S. A. Merchieva, M. A. Volodina, E. A. Sebentsova, D. M. Manchenko, V. S. Kudrun, N. G. Levitskaya

**Affiliations:** Institute of Molecular Genetics, Russian Academy of Sciences, Kurchatov Sq., 2, 123182 Moscow, Russia; Biological Faculty, Lomonosov State University, Moscow, Leninskie Gory, 1/12, Russia; Institute of Pharmacology, Baltyiskaya str, 8, 125315, Moscow, Russia

**Keywords:** biogenic amines, fluvoxamine, neonatal administration, psychomotor development, selective serotonin reuptake inhibitors

## Abstract

Selective serotonin reuptake inhibitors (SSRIs), including fluvoxamine, are widely used to treat depressive disorders in pregnant women. These antidepressants effectively penetrate through the placental barrier, affecting the fetus during the critical phase of neurodevelopment. Some clinical studies have linked prenatal exposure to SSRIs with increased neonatal mortality, premature birth, decreased fetal growth and delay in psychomotor development. However, the effects of prenatal exposure to SSRIs remain unknown. The administration of SSRIs in rodents during the first postnatal weeks is considered as an model for studying the effects of prenatal SSRIs exposure in human. The aim of this work was to study the acute effects of chronic fluvoxamine (FA) administration in white rat pups. The study was carried out in male and female rat pups treated with FA (10 mg/kg/day, intraperitoneally) from postnatal days 1 to 14. The lethality level, body weight, age of eye opening, and motor reflex maturation were recorded. The contents of biogenic amines and their metabolites in different brain structures were also determined. It was shown that neonatal FA administration led to increased lethality level, reduced body weight, and delayed maturation of motor reflexes. Furthermore, increased noradrenalin level in hypothalamus, serotonin level in hippocampus and serotonin metabolite 5-HIAA level in frontal cortex, hypothalamus, hippocampus, and striatum were observed in drug-treated animals compared to the control group. We can conclude that the altered activity of the serotoninergic system induced by fluvoxamine administration at early developmental stages leads to a delay in physical and motor development.

## INTRODUCTION

Depression is a common mental disease that occurs in more than 10% of the population. Women are much more susceptible to depression than men. Depressive symptoms are registered in 14–23% of women during the gestation period [1]. Selective serotonin reuptake inhibitors (SSRIs) have recently become the firstchoice medications for depressive disorders, and their use is constantly on the rise. This group of drugs includes fluoxetine, citalopram, fluvoxamine (FA), paroxetine, sertraline, and others. All SSRIs have a similar mechanism of action despite differences in their chemical structures [2]. The target of the action of SSRIs is the serotonin transporter (SERT) that is responsible for mediator reuptake from the synaptic cleft. SERT blockade increases serotoninergic neurotransmission. SSRI antidepressants are used to treat depressive disorders in pregnant and nursing women. According to various sources, 6–13% of women in their gestation period are currently taking SSRIs [3, 4]. Moreover, duration of intake and daily doses of the prescribed drugs increase [5]. SSRIs effectively penetrate through the placental barrier and are present in the amniotic fluid, umbilical blood, and fetal plasma [6]. The content of various antidepressants of this group in umbilical blood constitutes from 70 to 86% of the respective content in a mother’s blood; thus, the fetus is exposed to physiologically active SSRI doses [7]. However, the consequences of SSRI influence on a developing organism have been poorly studied so far. The results of clinical studies are extremely contradictory. Some works report no effect of drug exposure on the pregnancy complications and conditions of newborns [8-10]. Other data indicate negative effects from SSRI exposure on pregnancy outcomes: an increase in the number of spontaneous miscarriages and neonatal mortality, higher risk of premature birth, and a decrease in the birth weight were observed [1, 3, 11]. Symptoms of poor neonatal adaptation occurred in 15–0% of newborns prenatally exposed to SSRIs (neonatal withdrawal syndrome). Respiratory impairment, hypoglycemia, unstable body temperature, sleep disturbances, hyperexcitability, and convulsions are observed in infants during the first days of life. The indicated symptoms disappear during 1– weeks [1, 7]. Moreover, SSRI exposure during pregnancy (especially the last trimester) leads to a lower Apgar score of the newborns, delayed psychomotor development, sleep disturbances, persistent pulmonary hypertension, and cardiovascular disorders [2, 3, 6, 12, 13]. All the listed effects are observed in the early neonatal period (from birth to 6 months). Information about the delayed effects of prenatal SSRI exposure is limited due to time-consuming nature and complexity of such studies [13, 14]. As it has been previously mentioned, the results of clinical studies of prenatal SSRI exposure effects are rather contradictory. The reason for that may be the large heterogeneity of the pregnant women sampling used for the study. Women with different severity of depression, taking different drugs of the SSRI group in different doses and at different pregnancy stages, could be included in one group [11].

Effects of exposure to SSRI drugs on the developing brain are being actively studied in experiments on animals, mostly rodents. The third trimester of pregnancy is the period of human CN S development most sensitive to the action of SSRI [12]. It is very difficult to compare the development of human and rodent brains in the right way; however, data on CNS maturation (including the serotoninergic system) allow one to compare the last trimester of human pregnancy with the first weeks of life of rats [15, 16]. Therefore, SSRI effects during the first weeks of a rat’s life can be regarded as a model for studying the prenatal effects of this group of drugs during the third trimester of human pregnancy [17]. It was experimentally shown that chronic administration of SSRIs in the neonatal period causes long-term alterations in animal behavior. Adult rats and mice administered SSRIs during the first weeks of life displayed increased anxiety and depression, abnormalities in eating behavior, and alterations in the activity of the serotoninergic system [17, 18].

Thus, clinical studies of the effects of prenatal SSRI exposure are mostly focused on neonatal abnormalities, with data on the delayed effects of the exposure being limited. On the contrary, animal experiments are mostly aimed at estimating the long-term effects of perinatal SSRI administration [15]; studies of the neonatal effects are scarce [4, 19, 20]. However, studies of the acute effects of neonatal SSRI administration in animals are required to prove the adequacy of the experimental models in use.

*Fluvoxamine *is a modern antidepressant belonging to the SSRI group. Fluvoxamine is similar to fluoxetine in its pharmacological properties, but it is highly effective and selective [21] and possesses anxiolytic activity. The effects of neonatal FA administration have not been studied so far. In the present work, we have studied the influence of chronic neonatal fluvoxamine administration on the physical development and the state of the serotoninergic system in white rat pups.

## EXPERIMENTAL

Experiments were performed using both male and female pups of outbred white rats. The animals were kept under standard vivarium conditions with free access to food and water and a 12-h light regimen. The pups’ date of birth was considered postnatal day zero (PND 0). Two series of experiments were carried out.

The first series included 10 litters; the pups in each litter were divided into 3 groups: intact control (“IC”), control (“CON”) and fluvoxamine (“FA”). The use of the “IC” group was necessary to estimate the influence of everyday experimental manipulations on the observed parameters. No differences were found between the “IC” and “CON” groups in the first series of experiments: therefore, for 10 litters used in the second series of experiments each litter was divided only into 2 groups (“CON” and “FA”) in order to reduce the number of animals used. Rats from the “IC” group were subjected to everyday handling without drug administration from PND 1 to PND 14. Animals from the “CON” group were administered water for injections at 2 ml per kg of body weight by intraperitoneal (IP) injection, daily from PND 1 to PND 14. Rats from the “FA” group received IP injections of fluvoxamine (fluvoxamine maleate, Sigma) at 10 mg/kg of weight, daily from PND 1 to PND 14.

The age of eye opening and body weight of animals were registered in order to estimate the physical development of the pups. The level of psychomotor development was assessed in “righting reflex”, “gait reflex”, and “negative geotaxis reflex” tests. “Righting reflex”: a 6-day-old rat pup was placed in the supine position, noting the time needed for the animal to turn over (4 paws on the ground). “Gait reflex”: a 10-day-old rat pup was placed in the center of a circle 13 cm in diameter, noting the time needed for the animal to crawl out of the circle. “Negative geotaxis reflex”: a 12-day-old rat pup was placed on a 30-cm-long inclined surface (45°), with its head oriented towards the slope, noting the time needed for the animal to turn around, 180° [19, 20].

In order to study the effects of neonatal FA administration on the content of biogenic amines and their metabolites in a rat’s brain, some animals were decapitated at the age of PND 16 (48 h after the last injection). Brains were extracted with the following structures separated: frontal cortex, hypothalamus, hippocampus, and striatum. The specimens were rapidly frozen in liquid nitrogen and further stored at –0°C. Brain tissues were homogenized. High-performance liquid chromatography was used to determine the concentrations of biogenic amines and their metabolites, noradrenalin (NA), serotonin (5-HT), 5-hydroxyindoleacetic acid (5- HIAA), dopamine (DA), homovanillic acid (HVA), and 3,4-dihydroxyphenylacetic acid (DOPAC).

**Statistical data analysis**

The results were analyzed using the Statistica software package. The lethality levels in the groups were compared using the “difference between two proportions” test. Body weight data were analyzed by a two-way ANOVA for repeated measurements, with gender and group as between subject factors. The twoway ANOVA (gender × group) was used to compare the age of eye opening and psychomotor development of pups; post hoc testing was carried out by the LSD test. The content of biogenic amines in the brain was analyzed by a two-way ANOVA (gender × group or litter × group). The group means for normalized values of the contents of biogenic amines were compared using the Mann-Whitney test. The data in the figures are presented as the means ± standard error of the means. Differences were considered to be statistically significant with *p * < 0.05.

## RESULTS

The experiments were performed using both male and female animals. Application of the two-way ANOVA (factor 1 – group; factor 2 – gender) revealed no significant effect of the gender or interaction between the two factors in all the performed tests, allowing us to present the results obtained for the whole group of rats.

**Fig. 1:**
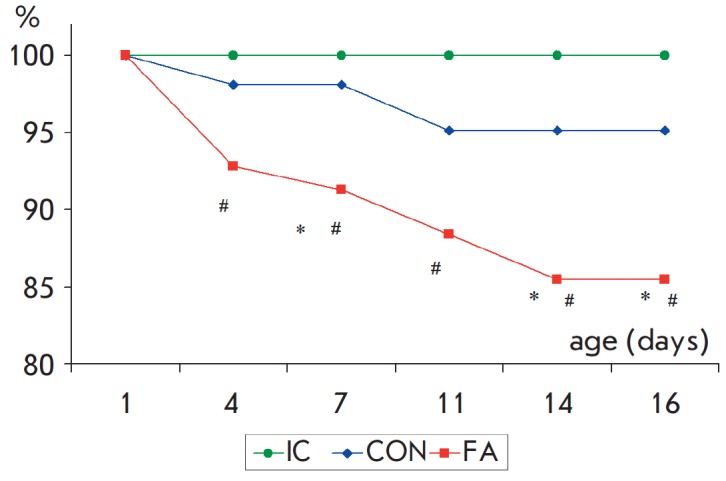
Effect of neonatal fluvoxamine administration on the lethality level in rats. X-axis – the age of rats, Y-axis – the number of survived animals as a percentage of the initial number of rats in the group (“IC” n=34, “CON” n=90, “FA” n=88). * –significant difference from the control, # –significant difference from “IC” group (difference between two proportions) (p < 0.05)

The lethality level in animal groups was estimated during the experiment. 100% of rats survived in the “IC” group by PND 16; 95.1%, in the “CON” group; and 85.5%, in the “FA” group (*Fig. 1*). Daily intraperitoneal injections of the solvent caused an increased lethality in the control group of rats; however, no statistically significant differences were observed compared to the intact control (*p *> 0.20). Chronic neonatal FA administration led to a significant increase in the lethality level compared to the control (*p* < 0.03).

**Fig. 2:**
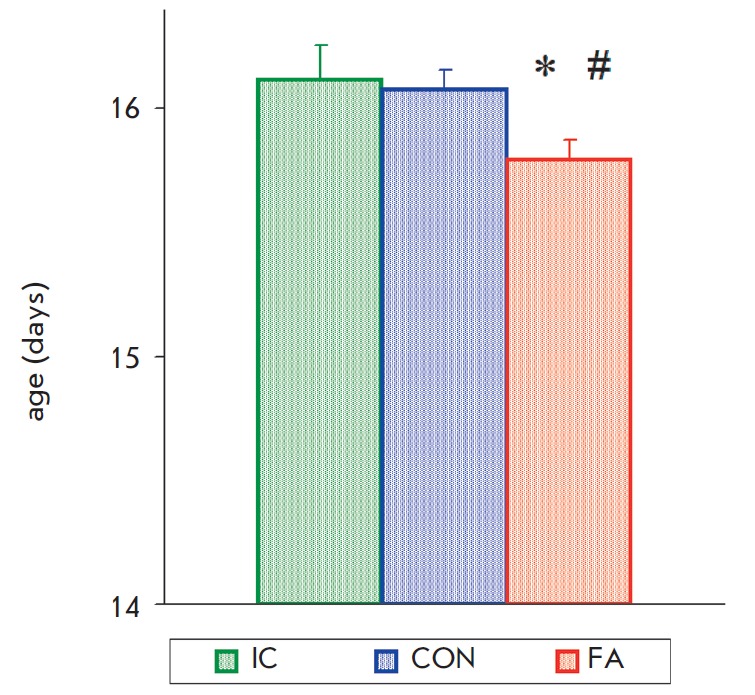
Effect of neonatal fluvoxamine administration on the age of eye opening (“IC” n=33, “CON” n=86, “FA” n=75). * –significant difference from the control, # –significant difference from the “IC” group (LSD test) (p < 0.05)

Analysis of the eye opening age in pups with neonatal fluvoxamine administration indicated no significant effect of gender (*F*_1,193_ = 2.73, *p *> 0.10) and statistically significant effect of group (*F*_2,193_ = 3.57, *p * < 0.03).. Post hoc analysis showed the presence of a slight but statistically significant decrease in the eye opening age in the “FA” group compared to the “IC” and “CON” groups (*Fig. 2*). 86.3% of the animals in the group of rats that received FA opened their eyes by the 16th day of life, as compared to 70.7% in the control group and 67.7% in the “IC” group (*p* < 0.03).

**Fig. 3:**
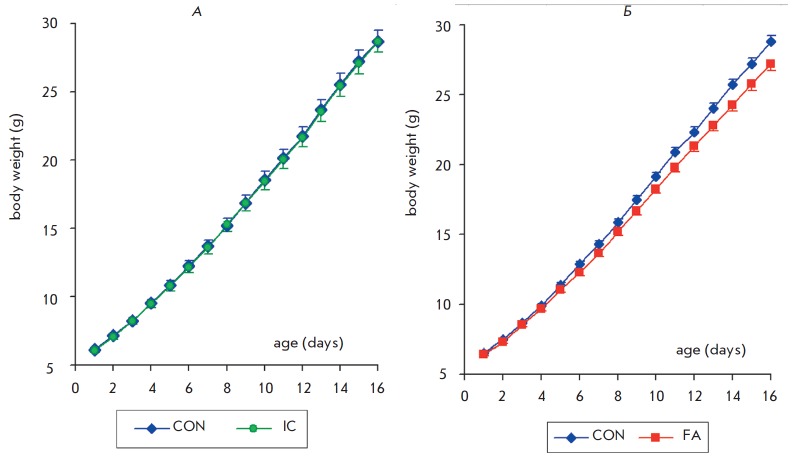
Effect of neonatal fluvoxamine administration on the weight gain in rats. X-axis – the age of rats, Y-axis – body weight (a – “IC” n=33, “CON” n=33, b –“CON” n=86, “FA” n=75)

The measurement of the body weight revealed statistically significant differences between newborn pups in the control groups in the first and the second experimental series (6.14 ± 0.13 and 6.50 ± 0.08 g, *p* < 0.01), while no initial differences between the “FA” and “IC” groups and the respective control groups were detected. *Fig. 3* shows the change in the rats’ body weight in the “FA” and “IC” groups as compared to the respective control groups. In all experimental groups, the body weight increased from PND 1 to PND 16 (*F*_15,2325_ = 4058.8; *p* < 0.001 and *F*_15,930_ = 1557; *p* < 0.001, *Fig. 3A* and *3B, *respectively). The effect of the gender on the body weight was not statistically significant in the first and the second experimental series (*F*_1,62_ = 0.70;* p *> 0.40 and *F*_1,155_ = 0.10; *p *> 0.80*, *respectively). Comparison of the “IC” and “CON” groups revealed no significant effect of the group on the body weight change in rats (*F*_1,62_ = 0.01; *p* < 0.98). In the case of “CON” and “FA” groups, a significant effect of the group on the weight gain was revealed (*F*_1,155_ = 4.1; *p * < 0.04). Therefore, daily intraperitoneal injections of the solvent did not affect the weight gain, while FA administration decelerated the growth of the animals.

**Fig. 4:**
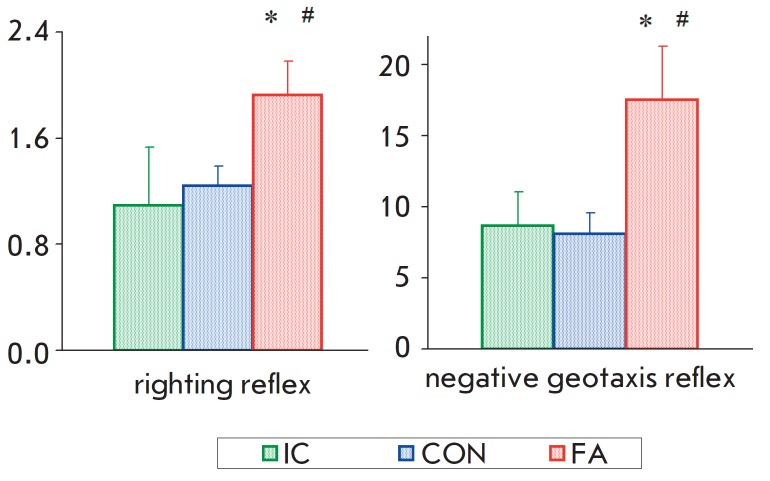
Effect of neonatal fluvoxamine administration on the latency time in righting reflex (PND 6; “IC” n=16, “CON” n=30, “FA” n=28) and in negative geotaxis reflex (PND 10; “IC” n=13, “CON” n=14, “FA” n=14). * –significant difference from the control, # –significant difference from “IC” group (LSD test) (p < 0.05)

No statistically significant effects of gender on the development of motor reflexes in the rats were observed (*F*_1,68_ = 0.17; *p *> 0.65 in the “righting reflex” test and *F*_1,35_ < 0.10; *p *> 0.80 in the “negative geotaxis reflex” and “gait reflex” tests). At the same time, the group significantly affected the response time in the “righting reflex” (*F*_2,68_ = 4.37; *p * < 0.04) and “negative geotaxis reflex” (*F*_2,35_ = 4.38; *p * < 0.05) tests, while there was no effect on rat behavior in the “gait reflex” test (*F*_2,35_ = 0.67; *p *= 0.52). Post hoc analysis revealed no significant differences between the “IC” and “CON” groups. The values of the registered parameters were statistically significantly higher in the “FA” group compared to the control and “IC” groups (*Fig. 4*).

**Fig. 5:**
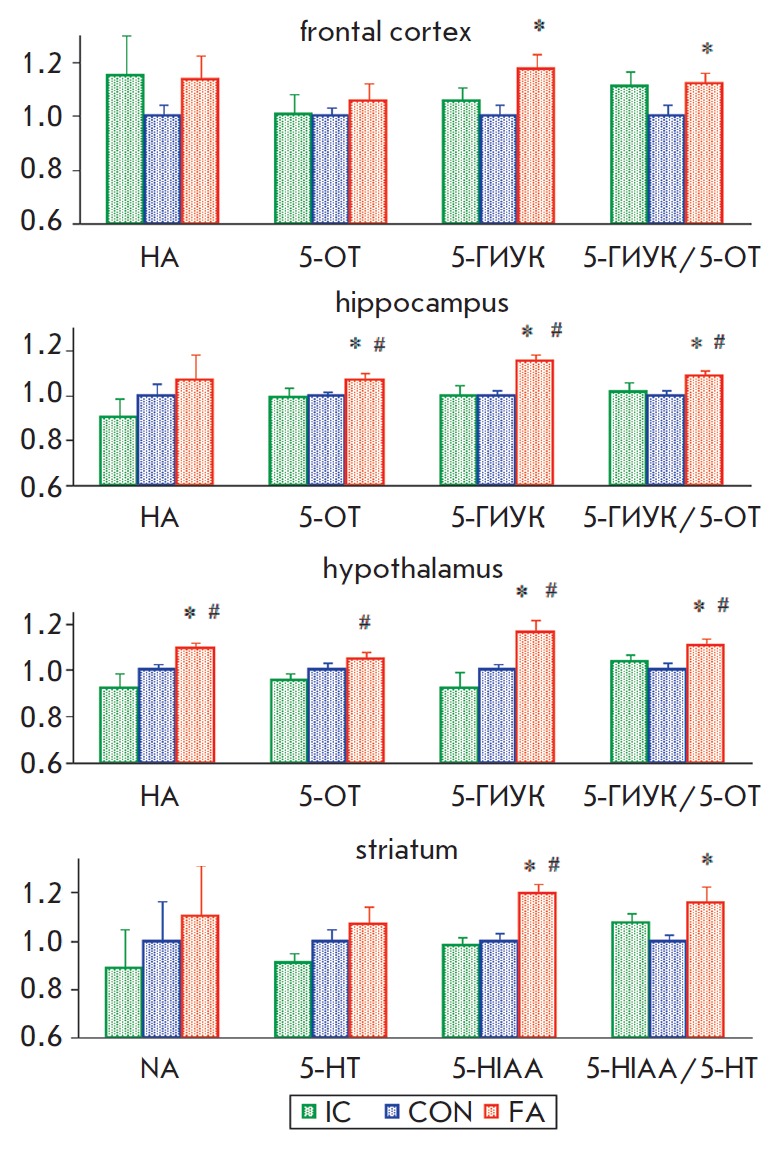
Effect of neonatal fluvoxamine administration on the contents of noradrenalin, serotonin and its metabolite 5-HIAA in different brain structures. Y-axis – parameter values normalized to the control (“IC” n=18, “CON” n=19, “FA” n=18). * –significant difference from the control, # –significant difference from the “IC” group (Mann-Whitney U test) (p < 0.05)

The contents of biogenic amines and their metabolites in a rat’s brain were measured on PND 16. The results are shown in *Table*. We observed no significant influence of gender on the registered parameters (*F * < 3.0; *p *> 0.10). Animals from five litters (3– rats from each group in every litter) were used for measurements. Application of the two-way ANOVA (factor 1 – group; factor 2 – litter) showed that the group significantly affected the following parameters: 5-HIAA content in hippocampus (*F*_2,38_ = 4.36; *p * < 0.02), frontal cortex (*F*_2,36_ = 3.55; *p * < 0.04) and striatum (*F*_2,40_ = 12.13;* p * < 0.001), as well as NA, 5-HT and 5-HIAA contents in hypothalamus (*F*_2,40_ > 4.5; *p * < 0.02). No significant influence of the group on the levels of DA and its metabolites was registered in any studied structure (*F * < 2.60;* p *> 0.10). Moreover, a significant influence of the litter on most parameters was noted (*F *> 2.95; *p * < 0.05), pointing to the variability of parameters in different litters. No significant interaction between the factors group and litter was observed (*F * < 1.50; *p *> 0.20). The values of the parameters for each litter were normalized to the respective controls to eliminate the influence of the litter. Further analysis revealed no statistically significant differences in the contents of biogenic amines and their metabolites in the studied brain structures between the “IC” and “CON” groups. In the “FA” group, a significant increase in NA content was observed in hypothalamus, as well as 5-HT content in the hippocampus and 5-HIAA content in all structures compared to the control (*Fig. 5*). 5-HIAA/5-HT ratio in the “FA” group in all structures was significantly higher than that in the “CON” group. A statistically significant increase in 5-HT contents in hippocampus and hypothalamus, NA content in the hypothalamus and 5-HIAA contents in hippocampus, hypothalamus and striatum, along with an increase in 5-HIAA/5-HT ratio in the hippocampus compared to the intact control were registered in the group of rats received FA. Moreover, a tendency towards increasing 5-HIAA level in the frontal cortex and increasing 5-HIAA/5-HT ratio in the hypothalamus and striatum compared to the intact control was observed (*p * < 0.10).

**Table:** Contents of biogenic amines and their metabolites (nmol/g tissue) in different brain structures

Biogenic amines and their metabolites	Frontal cortex				Hippocampus				Hypothalamus				Striatum			
	IC	CON	FA	F(p)	IC	CON	FA	F(p)	IC	CON	FA	F(p)	IC	CON	FA	F(p)
NA	0.40 ± 0.07	0.36 ± 0.02	0.39 ± 0.04	0.96 (0.39)	0.72 ± 0.11	0.76 ± 0.10	0.86 ± 0.12	0.56 (0.57)	3.10 ± 0.21	3.37 ± 0.10	3.63 ± 0.10	4.50 (0.02)	0.50 ± 0.14	0.67 ± 0.17	0.55 ± 0.14	0.48 (0.62)
DA	0.11 ± 0.01	0.18 ± 0.03	0.16 ± 0.03	2.26 (0.12)	0.05 ± 0.01	0.08 ± 0.02	0.05 ± 0.01	1.93 (0.16)	0.96 ± 0.07	1.21 ± 0.10	1.09 ± 0.06	2.63 (0.09)	19.93 ± 0.63	19.72 ± 0.76	18.74 ± 0.70	1.02 (0.37)
DOPAC	0.11 ± 0.04	0.07 ± 0.02	0.05 ± 0.02	1.64 (0.21)	0.17 ± 0.02	0.17 ± 0.02	0.16 ± 0.02	1.92 (0.16)	0.38 ± 0.05	0.35 ± 0.05	0.44 ± 0.04	0.74 (0.48)	3.46 ± 0.15	3.46 ± 0.16	3.31 ± 0.17	0.21 (0.81)
HVA	0.21 ± 0.04	0.19 ± 0.04	0.10 ± 0.02	2.37 (0.12)	0.54 ± 0.17	0.75 ± 0.12	0.75 ±0.16	0.65 (0.53)	0.37 ± 0.05	0.31 ± 0.04	0.35 ± 0.04	0.60 (0.55)	2.49 ± 0.20	2.56 ± 0.13	2.68 ± 0.11	0.25 (0.78)
5-HT	1.16 ± 0.09	1.16 ± 0.05	1.19 ± 0.08	0.40 (0.67)	1.22 ± 0.06	1.23 ± 0.03	1.30 ± 0.04	1.33 (0.28)	2.47 ± 0.15	2.92 ± 0.11	3.04 ± 0.11	8.33 (0.001)	1.15 ± 0.06	1.27 ± 0.06	1.36 ± 0.09	1.09 (0.35)
5-HIAA	0.44 ± 0.03	0.41 ± 0.03	0.47 ± 0.03	3.55 (0.04)	0.95 ± 0.07	0.96 ± 0.05	1.07 ± 0.06	4.36 (0.02)	1.49 ± 0.12	1.68 ± 0.09	1.89 ± 0.10	7.78 (0.002)	1.22 ± 0.08	1.32 ± 0.09	1.49 ± 0.09	12.13 (0.001)

## DISCUSSION

Comparison of the data obtained for the intact control and the control groups allows one to conclude that daily intraperitoneal injections of the solvent during the first 14 days of life do not lead to any statistically significant increase in the lethality level and do not cause significant changes in the rate of somatic growth, time of eye opening, and the development of motor reflexes, and therefore do not affect physical and sensomotor development of the animals. Moreover, daily injections of the solvent do not influence the state of the biogenic amine system in the brain of 16-day-old rats. Therefore, these experimental manipulations cause no significant changes in the physiological and neurochemical parameters registered in the present work.

A significant increase in the lethality level was noted in the group of rats that received daily FA injections compared to the control groups of animals. Moreover, a decelerated weight gain was observed in the “FA” group. Negative effects of neonatal administration of SSRI drugs on the change in body weight in the animals were registered in several studies. Thus, administration of citalopram [20], sertraline [19, 22], and fluoxetine [18] during the early development period in rats causes growth deceleration. The serotoninergic system is known to play an important role in the regulation of appetite and food consumption. Drugs that increase the extracellular 5-HT content also display marked anorexigenic activity [23]; therefore, it cannot be excluded that the SSRI effect on the weight gain is linked to the anorexigenic effects of 5-HT. However, it was also shown that neonatal SSRI administration leads to the development of the hypermetabolic state in mice [22]. The increase in the metabolism level in animals that received FA may also be a reason behind the decelerated weight gain.

We have also shown that rat pups that received fluvoxamine injections opened their eyes earlier compared to the control animals. Monoamines are known to act as trophic factors during the active development of the nervous system. During the prenatal and early postnatal periods, serotonin serves as a signal factor in cell proliferation and differentiation in the nervous tissue; it also influences the development of the epithelial tissue [15, 24]. It was shown that neonatal administration of the serotonin precursor 5-hydroxytryptophan leads to earlier eye opening [25]. It can be assumed that the increase in the activity of the serotoninergic system during this period accelerates the development of the visual analyzer. It is likely that this phenomenon explains the earlier eye opening in the group of rats receiving fluvoxamine injections.

We have registered an increase in the run-time for the righting and negative geotaxis reflexes in the group of rats receiving fluvoxamine injections, pointing to the deceleration in the maturation of motor reflexes. The delay in the development of motor reflexes was observed by Diero *et al*., who showed a delayed maturation of reflexes in the rats that were neonatally administered sertraline [19] and citalopram [20]. Therefore, SSRI administration to rats during the early postnatal period causes abnormalities in the development of motor functions. The changes in the content of biogenic amines caused by pharmacological or stress impacts during intensive brain development may lead to irreversible morphological and functional changes in the CNS [26]. Thus, neonatal fluoxetine administration decreases the number and size of 5-HT neurons in dorsal raphe nuclei and the quantity of 5-HT terminals in the hippocampus [27]. The increase in the serotonin content in the brain during the developmental period impairs axon myelination [28]. Neonatal SSRI impact causes morphological changes in neurons of the striatum and motor cortex: it reduces the length and branching of dendrites and the density of dendritic spines [17]. These changes may lead to the delay in the development of motor functions [28].

Our experiments showed that neonatal FA administration causes a deceleration of the somatic growth, decrease in eye opening age, and delay in the maturation of motor reflexes. The eye opening age and the changes in body weight reflect the level of animal’s physical development, while dynamic tests for the execution of motor reflexes allow one to estimate the maturation of the vestibular system. The multidirectional effects of neonatal influences on physical and motor development of animals was demonstrated in several studies. Thus, neonatal stress caused by long-term maternal deprivation leads to a decrease in the eye opening age and a delay in the maturation of motor reflexes. This was accompanied by an increase in the activity of the serotoninergic system in animals [29]. The influence of neonatal FA administration on the eye opening age is probably associated with its trophic function at the early stages of ontogenesis, since the acceleration of the development of nervous and epithelial cells may cause earlier maturation of the visual analyzer. The negative effect of FA on the maturation of motor reflexes can be determined by the morphological changes in CN S caused by neonatal SSRI administration. These changes impair the formation of connections between brain structures that may cause a delayed maturation of motor functions [28].

In our experiments, the levels of biogenic amines and their metabolites were measured 48 h after the last FA injection. Fluvoxamine has the shortest duration of action among all SSRI drugs; the half-life of this antidepressant is 15–17 h, while its metabolites exhibit no physiological activity [30]. Therefore, the withdrawal effects can be observed after 48 h. The experiments on adult animals showed that the content of serotonin metabolite 5-HIAA and 5-HIAA/5-HT ratio in various brain structures increased after discontinuation of chronic SSRI administration to rats [30–32]. Depending on the duration of the drug’s action, the effect develops 48–2 h after the last injection and persists for up to 2 weeks [33]. According to our data, discontinuation of FA administration to 14-day-old rats also increases 5-HIAA content and 5-HIAA/5-HT ratio in various brain structures. The 5-HIAA/5-HT ratio is an index of the serotonin turnover in the brain; an increase in this ratio indicates that the activity of the 5-HT system has increased.

According to the clinical data, an abrupt discontinuation of SSRI administration causes a withdrawal syndrome that includes the following symptoms: psychomotor agitation, anxiety, sleep disorders, vertigo, etc. The probable mechanism of this syndrome is an increase in the activity of the brain serotoninergic system [30]. Impairment of neonatal adaptation was noted for 15–30% of newborns who received SSRI prenatally [1]. Most researchers also attribute these impairments to the termination of the drug’s action [7, 11]. It can be assumed that the neonatal withdrawal syndrome is associated with the increased activity of the 5-HT system after termination of SSRI action, similarly to adult patients. Our data on the increase in the serotonin turnover rate in animals after the completion of the neonatal fluvoxamine administration course confirm this assumption.

Numerous clinical studies indicate that prenatal SSRI exposure (especially during the third trimester) negatively influence pregnancy outcomes and conditions of newborns. An increased number of spontaneous miscarriages and neonatal lethality, decreased birth weight, and further impairments of neonatal adaptation and delay in psychomotor development were noted [3, 11, 34]. The present work shows that chronic administration of the selective serotonin reuptake inhibitor fluvoxamine to white rat pups from the 1st to 14th days of life leads to an increase in the lethality level, deceleration of somatic growth, and delay in motor development. Moreover, increased activity of the serotoninergic system in response to the discontinuation of drug administration is observed in various brain structures. Our data allow us to conclude that SSRI administration to rat pups during the first weeks of life can be considered as an adequate model for studying the prenatal effects of this drug group in humans.

## Abbreviations

DA: dopamine
DOPAC: 3,4-dihydroxyphenylacetic acid
FA: fluvoxamine
5-HIAA: 5-hydroxyindoleacetic acid
5-HT: serotonin (5-hydroxytryptamine)
HVA: homovanillic acid
IC: intact control
NA: noradrenalin
PND: postnatal day
SERT: serotonin transporter
SSRI: selective serotonin reuptake inhibitors
